# 210. Evaluating the Costs of *Escherichia coli* Bloodstream Infections: A Population-Based Cohort Study

**DOI:** 10.1093/ofid/ofad500.283

**Published:** 2023-11-27

**Authors:** Teagan King, Ranjani Somayaji, Jenine Leal, Jason Black, Dan Gregson, John Conly, Elissa Rennert-May

**Affiliations:** University of Calgary, Calgary, Alberta, Canada; University of Calgary, Calgary, Alberta, Canada; University of Calgary, Calgary, Alberta, Canada; University of Calgary, Calgary, Alberta, Canada; University of Calgary, Calgary, Alberta, Canada; University of Calgary, Calgary, Alberta, Canada; University of Calgary, Calgary, Alberta, Canada

## Abstract

**Background:**

*Escherichia coli* (*E. coli*) is a frequent cause of blood stream infection (BSI) with associated morbidity and mortality. We evaluated the costs of susceptible and resistant *E. coli* BSI in children and adults. There is a paucity of studies examining *E. coli* BSI using micro-costing data within North America.

**Methods:**

Population-based cohort of all blood cultures from 2011 to 2018 in Calgary, Canada, linked to micro- and gross costing data using administrative databases. Compared no BSI to any *E. coli* infection, classified as resistant to any antimicrobial agent or susceptible, over 90 days. Propensity score matching was done 4:1 by age, sex, and comorbidities. The primary outcome was mean costs, using multivariable linear regression models in R version 4.1.0. Secondary outcomes were length of stay (LOS), readmissions and 90-day all-cause mortality. Comparative analyses were reported using odds ratios (OR) and 95% CIs.

**Results:**

A total of 37,482 children and 89,673 adults were included: 225 susceptible and 21 resistant BSI in children, and 3,870 and 711, in adults. Mean and standard deviation (SD) costs of an *E. coli* BSI including ED visits and hospitalizations for 90 days in children were $99,214 ($152,809) for susceptible, and $94,628 ($113,281) for resistant infections, and $36,104 ($74,274), and $57,162 ($176,019) in adults. The greatest cost difference in adults was resistant *E. coli* BSI compared to no BSI ($24,265, 95% CI $11,308-37,223, p< 0.001), with higher odds of readmission (OR 1.54, 95% CI 1.26-1.88, p< 0.001) and increased LOS (4.17 days, 95% CI 1.04-7.30, p=0.009). In children, cost differences were greatest for susceptible infections compared to no BSI ($45,186, 95% CI 27,965-62,406, p< 0.001), with the longest initial hospitalization (17.1 days, 95% CI 10.3-23.9, p< 0.001), and higher readmissions at 90 days (OR 1.65, 95% CI 1.17-2.31, p=0.004). *E. coli* BSI did not impact mortality for children or adults compared to those with no BSI.

**Conclusion:**

*E. coli* BSI is associated with substantial morbidity, mortality, and costs. Total cost differences were highest among adults with resistant, and children with susceptible infections compared to no BSI. Further research into cost-effectiveness of BSI diagnosis, treatment and prevention is warranted.

**Disclosures:**

**Ranjani Somayaji, M.D**, CF Foundation: Grant/Research Support|CIHR: Grant/Research Support|Oncovir: Data and Safety Monitoring Board Fees|Vertex Pharmaceuticals: Advisor/Consultant|Vertex Pharmaceuticals: Grant/Research Support|Vertex Pharmaceuticals: Honoraria **Dan Gregson, MD**, BioMerieux Canada: Advisor/Consultant

